# A Meta-Analysis of Dropout and Metabolic Effects of Antipsychotics in Anorexia Nervosa

**DOI:** 10.3389/fpsyt.2020.00208

**Published:** 2020-03-17

**Authors:** Carol Kan, Laura Eid, Janet Treasure, Hubertus Himmerich

**Affiliations:** Psychological Medicine, King’s College London, London, United Kingdom

**Keywords:** anorexia nervosa, dropout, antipsychotic, metabolic effect, meta-analysis

## Abstract

**Background:**

Second-generation antipsychotics are often used off-label in the treatment of anorexia nervosa (AN) across the clinical spectrum. Patients with anorexia nervosa often cite concerns about metabolic effects, such as weight gain, as reasons for their reluctance to start or continue second-generation antipsychotics. Improving our understanding of the metabolic effect patients experience and reasons underlying their disinclination will enable us to build rapport and guide our clinical decisions. We therefore aimed to conduct a comprehensive review of dropouts, metabolic effects, and patient-reported outcomes associated with second-generation antipsychotic in people with AN.

**Method:**

EMBASE, Medline, and PsycINFO were searched for all relevant studies published until 2019, and retrieved studies were assessed for eligibility as per predefined inclusion criteria. A random-effects meta-analysis was conducted to assess overall dropout rates.

**Results:**

Of 983 citations retrieved, 21 studies met the inclusion criteria for the systematic review and 10 studies had appropriate data for meta-analysis. Using the random effects model, the pooled dropout rate in the intervention arm (95% confidence interval) from psychopharmacological trials was 28% (19 to 38%) in people with AN. Personal reasons or factors associated with study were commonest reason for dropout, not adverse events or metabolic effects as hypothesized.

**Conclusion:**

Compared to personal reasons, drug-related factors such as side effects seem to play a lesser role for the discontinuation of antipsychotic treatment under trial conditions. This suggests an urgent need to consider and fully examine potential individual and patient-related factors that influence dropout rates in psychopharmacological trials and treatment compliance in clinical settings.

## Background

Anorexia nervosa (AN) is a debilitating illness with a median duration of 7–10 years. Approximately 20% of individuals with AN develop a severe and enduring form of the illness (1) and a 22 years follow-up study found that only 68.2% of participants with AN have recovered (2). Treating AN successfully is a challenge. Admissions have continued to rise exponentially in the UK (3), even though National Institute for Health and Care Excellence (NICE) recommended outpatient treatment as a first line of management since 2004 (4, 5).

Various forms of outpatient psychological therapies have been developed. A recent meta-analysis of 35 randomized controlled trials (RCTs) concluded that specialised AN treatment was not superior to standard treatment at follow-up [effect size (95% CI) for i) for weight outcomes: 0.11 (-0.04 to 0.27) and ii) psychological outcomes: -0.001 (-0.11 to 0.11)] (6). A recent Cochrane Review also reported that there is little or no difference between specialist inpatient care and active outpatient or combined inpatient and outpatient care in weight gain at 12 months after the start of treatment [standardised mean difference (SMD): -0.22 (-0.49 to 0.05)] (7).

Second-generation antipsychotics are often used off-label in the treatment of AN. For example, a recent self-reported survey of prescribing practice in the UK reported that psychotropic medications are commonly prescribed to a minority of children and adolescents with AN, with olanzapine being the most frequent psychotropic drug (8). The rationale for their use includes weight gain and symptom relief such as reducing anxious feelings and weight/shape concerns. The clinical effectiveness of antipsychotics for people with AN is, however, unclear at present. To date, there have been four meta-analyses examining this effect (9–12). None of the meta-analyses reported a statistically significant differences for weight or changes in body mass index (BMI) between antipsychotics and active control/placebo groups.

The metabolic effects of second-generation antipsychotics are serious and wide-ranging. They have been associated with weight gain, dyslipidemia, hypertension, hyperglycemia, and type 2 diabetes in people with schizophrenia (13). Genetic vulnerability may be relevant in developing metabolic effects. A recent genome-wide association study reported significant genetic correlations between metabolic-related phenotypes with AN, with the authors encouraging a reconceptualization of AN as a metabo-psychiatric disorder (14). This concept is further supported by a meta-analysis of clinical studies showing that AN is associated with increased insulin sensitivity [effect size (95% CI): 1.66 (0.79 to 2.54)].

Patients with AN often cite metabolic effects as reasons for their reservations towards second-generation antipsychotics (15). For example, weight gain is a desired side effect for AN from a clinician's perspective, but patients are often reluctant to start or continue with them. Drop-out rate in clinical trials is defined as the proportion of participants who begin, but do not complete the full course of recommended treatment. It is a useful metric in determining the tolerability of a treatment. We therefore conducted a meta-analysis on the drop-out rates from psychopharmacology trials with second-generation antipsychotics. It can serve as a proxy indicator of the likelihood of a patient with AN in continuing second-generation antipsychotics. We also explored self-reported side effect, as patient-reported outcomes provide a unique window in directly understanding patients’ experience and guide clinical decision-making (16). The main aim of this review is therefore to conduct a comprehensive review of dropouts, metabolic effects, and patient-reported outcomes associated with second-generation antipsychotic in people with AN.

## Method

### Eligibility Criteria

Abstracts were considered eligible for full manuscript data extraction if the study met all the following criteria: i) they compared the treatment outcomes of second-generation antipsychotics for AN; ii) the design was RCTs, open label trials, or observational studies; and iii) published in English. Participants of any age and any sex, with a diagnosis of AN were considered. For the meta-analysis, only studies with information about the proportion of participants who begin but do not complete the full course of recommended treatment in the intervention arm were considered.

### Study Selection

We searched the Embase (1985 to 2019), PsycInfo (2005 to 2019), and Medline (1982 to 2018), using Ovid Database. We also hand-screened reference lists of all included articles to identify additional studies that were not found during the initial search. Keywords used in the searches included, but not limited to “anorexia nervosa”, “atypical antipsychotic”, “second generation antipsychotic”, “amisulpride”, “aripiprazole”, “asenapine”, “brexpiprazole”, “cariprazine”, “clozapine”, “lurasidone”, “olanzapine”, “paliperidone”, “pimavanserin”, “quetiapine”, “remoxipride”, “risperidone”, “sertindole”, “ziprasidone” (exhaustive list of keywords in Appendix 1).

### Data Extraction

Using a standardized data extraction sheet, the following information (if available) was extracted and recorded for each study: authors; year of publication; country of origin; treatment setting; study design; duration of intervention; age; sex; total numbers of participants in each treatment group; drop out in intervention group; reasons for dropouts; adverse event reporting; duration of intervention and concurrent treatment.

Dropout was defined as the number of participants who started treatment and were defined as having dropped out according to the definition of the study. We did not distinguish the time point of the dropout, as participants can drop out prior to starting the intervention, during the intervention, and after completing the intervention but fail to attend follow-up. The metabolic effects considered included but not limited to weight/BMI, glycaemic control, electrocardiogram (ECG) reports, lipids profile, liver function tests, prolactin, and self-reported side effects.

### Quality of Study

To assess the quality of studies, the Cochrane Collaboration's tool for assessing risk of bias was applied to randomized trials and Risk Of Bias In Non-randomized Studies-of Interventions (ROBINS-I) assessment tool for non-randomized studies (17, 18). These include adequacy of study design (observational or open label trails or RCT with an adequate control group); recruitment of sample and control for cofounding variables, such as age, sex, socioeconomic status. A study was considered to be of high quality if the study design consists of a control group; consecutive or random sampling method was used; adequate blinding of participants and personnel; appropriate reporting of outcome variables.

### Statistical Analysis

All statistical analyses were performed using the “Metafor” package (19) in open source software programme R. We first transformed the proportion data using the Freeman-Tukey (double arcsine) transformation. This approach is recommended for proportion data as it produces more stable estimates of corresponding sampling variances for the sampling distribution of proportions close to 0 or 1 (20, 21). The transformed proportions and corresponding sampling variances were used in the meta-analysis and then back-transformed using the equation derived by Miller (22) for ease of interpretation.

To estimate the average overall dropout rate from active treatment, a weighted pooled event rate was calculated (“escalc” function in “Metafor”). The meta-analysis was conducted using a random effects model. Given the likely significant differences in study design, a fixed-effects model was deemed inappropriate. Significance threshold was set at p < 0.05. A Cochran Q test was performed to assess between-study heterogeneity of effect size (23). Publication biases were investigated with visual inspection of the funnel plots and Begg's rank correlation tests for funnel plot asymmetry (24).

## Results

### Study Selection

**Figure 1:**
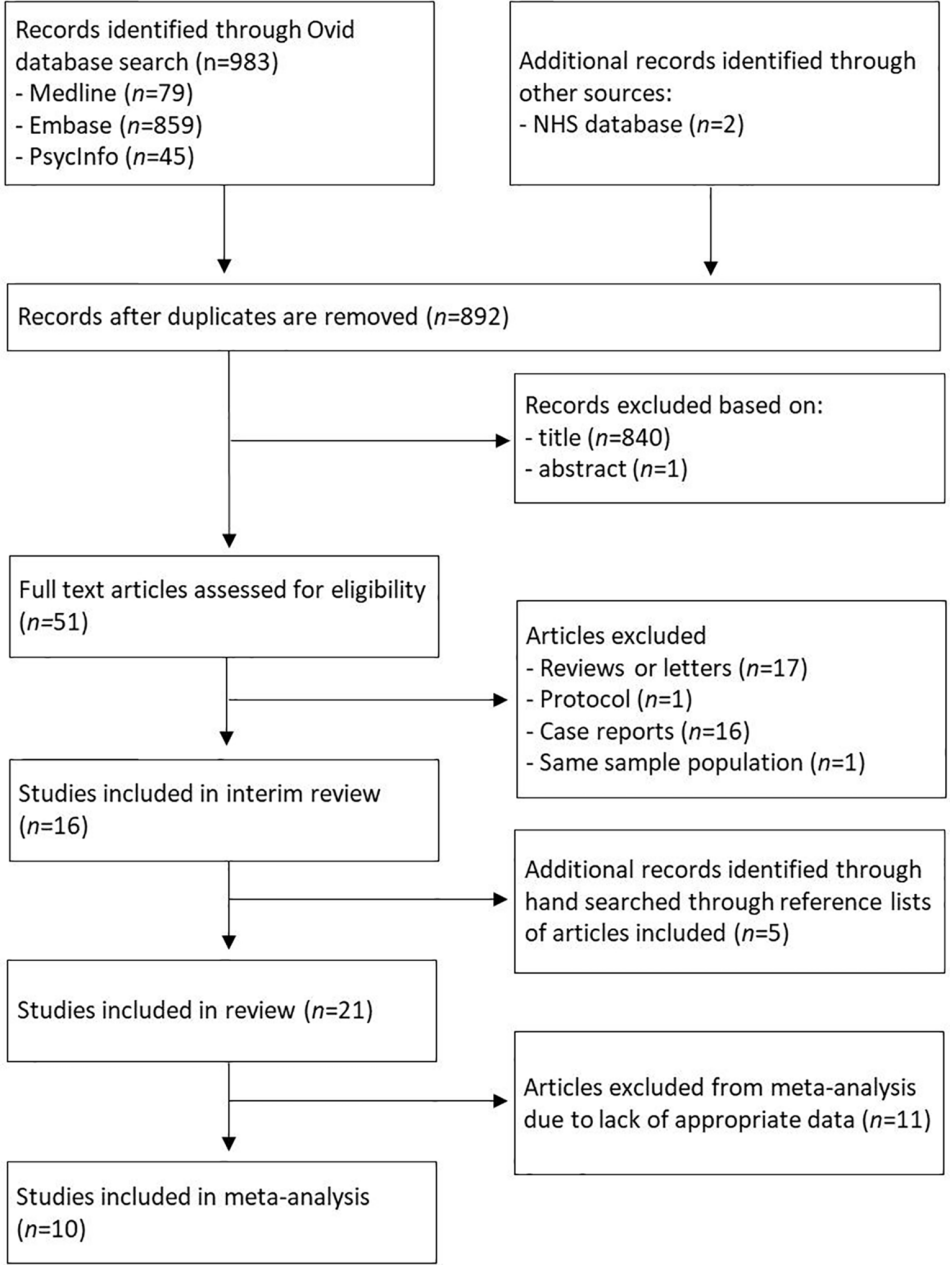
Flow chart of studies included in systematic review.

The literature search resulted in 983 studies (**Figure 1**). After reviewing their titles and abstracts, 51 studies met the inclusion criteria and were retrieved for full text. Of these, 33 studies were excluded from the systematic review as they did not meet the inclusion criteria. Upon closer inspection of the remaining 18 studies, two studies utilized the same study population (25, 26). The study with the main aim of reporting the findings of olanzapine in people with AN was selected (25). One study was published as an abstract and the journal is now defunct (27). We therefore did not have access to any information about sampling method, baseline clinical characteristics of the sample population and variables of interest. Five additional studies that fulfilled the inclusion criteria were found from searches of the reference lists of included articles. In total, 21 studies were included in the systematic review and summarised in **Table 1**.

**Table 1:** Summary table of primary studies included in the systemic review.

Author	Setting	Study type	Adults/Adolescent Age mean (SD)	% of female	Number of participants	Dropout in intervention group	Reasons for dropout	Adverse event	Intervention dosage mean (SD)	Duration of intervention(weeks)	Concurrent treatment
*Attia (28)	US/Canada; Outpatient	RCT	Adults Cx: 30.0 (11.0) Ix: 28.0 (10.9)	96 (146)	Cx: 77 Ix: 75	34/75	Hospitalisation: 5 Side effect: 4 Suicidal ideation: 3 Other: 1 Voluntarily withdrawal: 12 Lost to follow-up: 9	Hospitalisation: 5 Suicidal ideation: 3	Olanzapine 7.77 (1.07)	16	Psychotropics (41.4% in sample)
Spettigue (29)	Canada; Mainly Inpatient	Open label trial	Adolescents 15.48 (1.45)	90.6 (29)	At baseline, Cx: 18; Ix: 14 8 participants switched to Ix At end of study, Cx: 10; Ix: 22	NA	Incapable to participate: 1 Unable to adhere to protocol: 1 Preferred a different Ix: 4	None	Olanzapine 5.28 (NA)	12	Individual and family therapy
Frank (30)	US; Inpatient/ partial inpatient	Retrospective chart review	Adolescents Cx: 14.4 (2.5) Ix: 15.0 (2.2)	NA	Cx: 84 Ix: 22	NA	NA	NA	Aripiprazole 3.59 (1.85)	Minimal 3	Specialized eating disorders program
Marzola (31)	Italy Inpatient	Retrospective chart review	Adults 25.43 (9.4)	90.7 (68)	Cx: 25 Ix-A: 23 Ix-B: 27	NA	NA	None	Ix-A: Aripiprazole 9.13 (6.33) Ix-B: Olanzapine 6.11 (3.27)	Mean: 4.96 (1.62)	Antidepressants
*Powers (32)	US; Outpatient	RCT	Adults 34 (13.48)	93.3 (14)	Cx: 9 Ix: 6	2/6	Hospitalisation: 2	Hospitalisation: 2	Quetiapine 177.7 (90.8)	8	NA
Attia (33)	US/Canada; Outpatient	RCT	Adults 27.7 (9.1)	95.7 (22)	Cx: 12 Ix: 11	NA	NA	None	Olanzapine 7.95 (2.7)	8	Antidepressants; no psychological therapy
*Hagman (34)	US; Inpatient, outpatient and daycare	RCT	Adolescents 16 (NA)	100 (40)	Cx: 22 Ix: 19	3/19	Requested active Ix: 1 Reasons unrelated to study: 2	None	Risperidone 2.5 (1.2)	Mean: 8.6 (4.2)	Antidepressants (40% in sample)
*Kafantaris (35)	US;Inpatient, outpatient and daycare	RCT	Adolescents 17.1 (NA)	100 (20)	Cx: 10 Ix: 10	3/10	Sedation: 1 Stopped Ix: 2	None	Olanzapine 8.5 (NA)	10	Comprehensive eating disorders programme; no group/family therapy
Norris (36)	Canada;Inpatient, outpatient and daycare	Retrospective	Adolescents	100	Cx: 43	NA	NA	None severe	Olanzapine	Minimal 2	Antidepressants (39%) +/-
		cohort study	Cx: 14.8 (1.6) Ix: 14.4 (1.9)	(86)	Ix: 43				5.0 (3.75- 7.5)	In subgroup	benzodiazepine (7%)
*Court (37)	Australia; Outpatient and inpatient if needed during stay	Open label RCT	Adults 22.53 (NA)	97.0 (32)	Cx: 18 Ix: 15	5/15	Withdrawn consent: 1 Adverse event: 1 Side effect: 1 Non-response: 1 Failure to attend: 1	None severe	Quetiapine 322.5 (NA)	12	Antidepressants +/- benzodiazepine
Leggero (38)	Italy; Outpatient	Prospective cohort study	Adolescents 13.7 (2.3)	NA	Ix: 13	NA	NA	NA	Olanzapine	Unclear	Multimodal including psychotherapy, assisted feeding, psychoeducation, and prolonged control of somatic conditions
*Bissada (39)	Canada; Daycare	RCT	Adults Cx: 29.67 (11.59) Ix: 23.61 (6.50)	100 (34)	Cx:18 Ix: 16	2/16	Discontinued daycare: 1 Stopped Ix: 1	None	Olanzapine 6.61 (2.32)	10	Comprehensive day programme Including group therapy
Brambilla (25)	Italy; Outpatient	RCT	Adults Cx 26.3 (8.5)	100 (30)	Cx: 15 Ix: 15	NA	NA	NA	Olanzapine 7.77 (1.07)	12	CBT
*Bosanac (40)	Australia; Inpatient/outpatient	Open label trial	Adults 33.25 (7.65)	100 (8)	Ix: 8	3/8	Own request: 1 Substance misuse: 2	None	Quetiapine 520 (277.49)	8	Specialised and multidisciplinary treatment
*Powers (41)	US; Outpatient	Open label trial	Adults 26.8 (11.2)	94.7 (18)	Ix: 19	5/19	Unknown: 1 Seek treatment elsewhere: 1 Weight gain: 1 Moved out of area: 1 Non-response: 1	None severe	Quetiapine 150-300 (NA)	10	None
Mondraty (42)	Australia; Inpatient	RCT	Adults	NA	Cx: 7	NA	NA	NA	Cx: Chlorpromazine	Mean:	Antidepressants
			25.3 (NA)		Ix: 8				50 (NA)	(Unit: days)	(63% in Ix arm)
									Ix: Olanzapine	Cx: 53 (26)	
									10 (NA)	Ix: 46 (31)	
*Barbarich (43)	US; Inpatient & outpatient	Open label trial	Adults 20.5 (5.1)	NA	Ix: 17	5/17	NA	NA	Olanzapine 4.7 (1.6)	6	CBT and DBT mainly, with 1 participant being on antidepressants
Malina (44)	US; Inpatient	Retrospective	Adults	NA	Ix: 18	NA	NA	NA	Olanzapine	Range:	Antidepressants,
		cohort study	22 (7)						4.7 (2.4)	17-20	benzodiazepine
*Powers (45)	US; Outpatient	Open label trial	Adults 26.8 (12.3)	88.9 (16)	18	4/18	Hospitalisation: 1	NA	Olanzapine 10 (NA)	10	Psychotherapy for 1 participant
											and benzodiazepine for 2 participants
Ruggiero (46)	Italy; Inpatient	Single blind RCT	Adults Cx-A: 23.69 (4.57) Cx-B:	NA	Cx-A: 13 Cx-B: 10 Ix: 12	NA	NA	NA	Cx-A: Clomipramine 57.69 (25.79) Cx-B:	12	Weight gaining programme
			24.5 (50.6)						Fluoxetine		
			Ix: 24.33 (5.76)						28 (10.32)		
									Ix: Amisulpride		
									50 (NA)		
Beasley (47)	US; Inpatient & outpatient	RCT	Adults 38	NA	Cx: 50 Ix-A: 52 Ix-B: 50	NA	NA	SCZ worsen: 1 Hyponatraemia: 1 Hypotension: 1 Urticaria: 1 Allergic reaction: 1 PD: 1	Ix-A: Olanzapine 1 Ix-B: Olanzapine 10	6	Benzodiazepam and anticholinergic agent

NA, Not Available; SD, Standard Deviation; Ix, Intervention Group; Cx, Comparison Group; RCT, Randomized Controlled Trial; CBT, Cognitive Behavioural Therapy; DBT, Dialectic Behavioural Therapy; SCZ, Schizophrenia; PD: Personality Disorder.

*Studies included in meta-analysis.

11 studies were excluded from the meta-analysis because they did not report information about the proportion of participants in the intervention arm who did not complete the study. Raw data were therefore not available to generate a standardized effect size for dropout rates. This resulted in 10 datasets being included in the meta-analysis.

### Qualitative Summary

Of the 21 datasets included in the systematic review, 11 were RCTs (25, 28, 32–35, 39, 42, 46, 47), 5 open label trials (29, 40–43, 45), 1 prospective study (38) and 4 retrospective cohort studies (31, 36, 44). Sample size varies substantially, with a range between 8 and 152. The median number of participants was 31 with an interquartile range of 18 and 39. There were only 3 studies with a sample size over 100 (106, 152, and 152 respectively). In addition, the majority of the studies included in the systemic review (*n* = 10) have been conducted in USA, with the rest being in Canada only (*n* = 3), Canada/US (*n* = 1), Australia (*n* = 3), and Italy (*n* = 4). In terms of sample populations, 6 studies focused on adolescents, with the mean age ranging from 13.7 to 17.1, while 15 studies focused on adults, with a mean age ranging from 20.5 to 38. Of the 14 studies with information on gender, 6 studies recruited only females, while the proportion of females in the remaining 8 studies range between 88.9 and 97.0%.

In terms of treatment setting, 6 studies were conducted in inpatient, 7 in outpatients, 1 in day-care, and 7 across all three settings. For concurrent treatment, 20 studies reported adequate information for extraction. 10 studies allowed for concurrent medication, mainly antidepressants and benzodiazepines, 3 studies combined psychotherapy with second generation-antipsychotics and 6 studies was part of a comprehensive eating disorder treatment programme or its participants receive multidisciplinary team inputs which can include psychotherapy and meal support. One study recorded that no participants received psychotherapy or any other psychiatric treatment during the study. For second-generation antipsychotics as the study drug in the intervention group, 13 studies examined olanzapine, 4 quetiapine, 1 risperidone, 1 aripiprazole, 1 amisulpride, and 1 study compared olanzapine with aripiprazole. Their associated treatment duration ranges from a minimum of 2 weeks to a maximum of 20 weeks. Descriptive data from the datasets are summarized in **Table 1**.

For the meta-analysis, 7 were RCT (25, 28, 32, 34, 35, 37, 39) and 3 open label trials (40, 41, 45). Total number of participants in the intervention group ranged between 6 and 75. The majority of the studies have been conducted in USA (*n* = 6), with the rest being in Canada only (*n* = 1), Canada/US (*n* = 1) and Australia (*n* = 2). Two studies focused on adolescents, with the mean age ranging from 16 to 17.1. 8 focused on adults, with a mean age ranging from 20.5 to 34. In terms of second-generation antipsychotics as the study drug in the intervention group, 5 studies examined olanzapine, 4 quetiapine and 1 risperidone.

### Meta-Analysis

The pooled dropout rate in the intervention arm was estimated to be 28% (95% CI: 19 to 38%; p < 0.01) in a random effect model. Results were not statistically significant heterogeneous (Q: 11.96, df: 9, p: 0.02, I^2^: 33.1%). Visual inspection of the funnel plot suggested asymmetry, but the Begg's rank correlation test was statistically insignificant (τ: 0.27, p: 0.28), indicating no publication bias.

### Outcome of Interest: Dropout

Ten studies specified reasons for dropout. The percentage of each dropout reason, as a proportion of all the reasons given, are described below from most prevalent to least prevalent. The commonest reason for dropout was personal choices (43.5%; 27/62), such as participants' voluntarily withdrawal from a study or preference of a different intervention. This was closely followed by study reasons, for example being lost to follow-up or failure to adhere to protocol (25.8%; 16/62). Of interest, 16.1% (10/62) of dropouts were due to poor response to the intervention or deterioration in mental state, with 6 participants requiring hospitalization during the study. Adverse events were cited as a reason for dropouts in only 12.9% cases, with weight gain being mentioned once. There were, however, three incidences of suicidal ideations (4.8%) as a reason for dropout from one study (28).

### Outcome of Interest: Metabolic Effects

All studies included in the systematic review provided some information for weight/BMI/ideal body weight. This information was, however, difficult to interpret due to heterogeneity between studies. This included different duration of intervention and measures of weight, with some focus on weight gain over time or changes in pre- and post-intervention BMI between groups. In addition, the handling of missing data analysis was often not discussed, leading it hard to summarize the results in a systematic manner. In brief, 6 studies reported a statistically difference between intervention and comparison groups in either weight/BMI increase over time (28–30, 33, 36, 47) whereas 9 studies concluded the opposite (25, 32, 34, 46, 47). Six studies focused on the intervention group only, with 4 reporting a statistically significant weight gain/BMI increase or a greater proportion reaching their ideal body weight (38, 40, 43, 45). One study did not observe any statistically significant change in weight between baseline and end of treatment (41) while one study did not report any significance testing (44).

For glycaemic control, seven studies reported their findings. One study reported a statistically significant increase in fasting glucose in the intervention group (olanzapine) between baseline and end of study (35) whereas one study reported that 4 participants reported hypoglycaemic events with olanzapine (42). For ECG results, four studies reported their findings. One study reported that QTc prolongation was observed in one participant from both intervention (olanzapine) and comparison groups (29) whereas another study reported that one participant developed borderline QTc prolongation with olanzapine (31).

For other routine metabolic effects, there was limited information, with some studies stating that no abnormalities were observed during routine laboratory investigation. In summary, 5 studies reported lipid profiles, 5 for liver function test, and 2 on prolactin. Most studies did not report any statistically significant difference between intervention and comparison groups. One study reported that 3 participants developed raised cholesterol and one participant developed raised low-density lipoprotein in the intervention group (olanzapine) during the study period (31). One study concluded that more participants in the intervention group (olanzapine) experienced clinically significant abnormalities in lipid profiles, liver function tests and prolactin than comparison group, but the differences did not reach statistical difference (29). Another study reported that there is statistically difference in prolactin level between intervention (risperidone) and comparison groups (34), while another study reported that two participants in one study developed raised liver enzymes with olanzapine (38).

### Outcome of Interest: Patients Reported Physical Side Effects

Second-generation antipsychotics are associated with a range of physical side effects which may not be captured by biometric investigations. All studies included in the systemic review were examined for any description of patient reported physical side effects. In brief, 14/21 (67%) studies documented physical side effects or adverse events. There was no information on the duration of the side effects, except for two studies describing them as mainly transient (37, 41).

In the intervention group, the most common side effect was sedation which was reported in 12 out of the 21 studies (57%). This was followed by dizziness (n = 7 studies, 33%), headache (n = 7, 33%), gastrointestinal problems (n = 6, 29%), insomnia (n = 5, 24%), fatigue (n = 4, 19%), muscular problems (n = 4, 19%). A few studies also reported dry mouth (n = 3, 14%), blurred vision (n = 2, 10%), poor concentration (n = 2, 10%), agitation (n = 2, 10%), respiratory problems (n = 2, 10%), lower extremity oedema (n = 2, 10%), difficulty in staying still (n = 1, 5%), paraesthesia (n = 1, 5%), and dental problems (n = 1, 5%). Of interest, participants who received placebo also reported physical side effects (n = 3, 14%) (28, 29, 34). These included troubles concentrating, subjective restlessness, agitation, sleep problems, headache, constipation, dizziness, muscle stiffness, somnolence, dry mouth, fatigue, dizziness, gastrointestinal complaints, and headache.

### Risk of Bias and Strength of Evidence Quality

**Figure 2:**
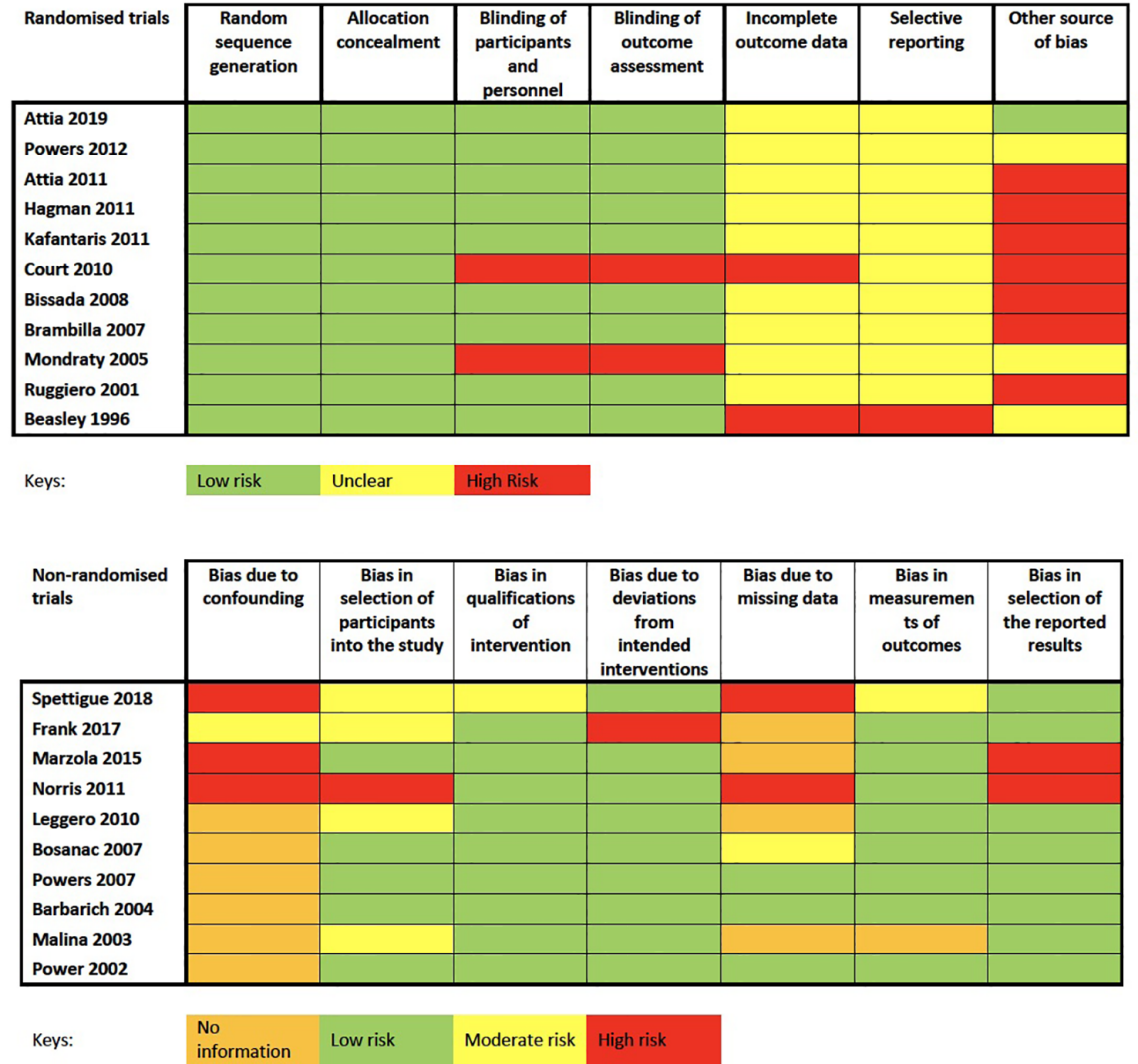
Cochrane Collaboration's tool for assessing risk of bias for randomized trials and Risk of Bias in Non-randomized Studies-of Interventions (ROBINS-I) assessment tool for non-randomized studies.

For randomized trials, two studies did not blind participants and personal adequately (37, 42). For non-randomized trial, six studies did not have a comparison group as they were observation studies (25, 38, 40, 44, 45). Other sources of bias were which could potentially threaten the external and internal validity of the findings include heterogeneous demographics across groups during baseline, a short duration of treatment, wide range of medication dosage, or confounding variables such as concurrent treatment. In addition, sample sizes of each individual studies were generally small and of the 21 studies included in the systematic review, only 10 studies reported appropriate dropout rates for the meta-analysis, further reducing the sample sizes. The challenges of small sample size were further compounded by the significant dropout observed and only missing data analysis was often not clearly detailed. The overall risk of bias was therefore low to medium (**Figure 2**).

## Discussion

To our knowledge, this is the first systematic review and meta-analysis of dropout associated with second-generation antipsychotics in people with AN. Our finding of 28% dropout rate (95% CI: 19 to 38%) from psychopharmacological trial is low when compared to 48.1% reported in a meta-analysis of second-generation antipsychotics in people with schizophrenia or schizoaffective disorder (48). Our dropout rate is, however, high when compared to psychotherapy. Meta-analyses have reported dropout rates of 24% for cognitive behaviour therapy in people eating disorders (49), 18.7% for interpersonal therapy in eating disorders (50) and 19.9% for individual psychotherapy in unipolar depression (51). Of interest, personal choices and factors associated with individual trials are the main reasons for dropout, not metabolic effects such as weight gain as initially hypothesised. Thus, our findings prompt the need to consider how specific patient and study characteristics may influence dropout rates in psychopharmacological trials in people with AN.

It is important to interpret our findings in the context of difficulties of recruiting and retaining participants with AN in psychopharmacological studies. For example, in a study of quetiapine, only 10.4% of the adult patients (21/217) consented to treatment (32). A study in adolescents examining risperidone was ended after 4 years due to slow recruitment and exhausted funding (34). Thus, the extent of generalizability of our findings to those who declined to participate is at present unclear.

### Limitations

A major source of heterogeneity is study design, given only 11 out of the 21 studies included in the systematic review were RCT in design. Another source of heterogeneity is concurrent treatment and study setting. The regimen of the second-generation antipsychotics also varies substantially between and within studies. The dosage is often flexible and individual-tailored within studies. A major limitation of our review is that most studies included in the systematic review (67%) and meta-analysis (80%) were from USA/Canada, rendering it difficult to generalize our findings. In addition, most studies were small in size. Another major limitation of our review is that most studies included were small in size. This is further compounded by the significant proportion of participants who did not complete a study. Thus, adopting the “intention-to-treat” in data analysis according to the treatment groups being assigned at randomization, with appropriate handling of missing data is important. In addition, the long-term metabolic effects of second-generation antipsychotics in people with AN is currently unclear, as only two studies discussed follow-up beyond 3 months (37, 38).

### Clinical Implications

Recruitment and retainment rates are a major challenge for psychopharmacology studies in AN. To encourage patients to consider second-generation antipsychotics as part of their treatment plan, it is important to explain the potential side effects experienced and stress the transient nature. Given that adverse events such as suicidal ideation were observed (28), it is important that patients are reviewed after being commenced on second-generation antipsychotics and address any side effects experienced promptly. Including family members in discussions about treatment and potential risks can allow for a more balanced appraisal of possible costs and benefits.

## Conclusion

Our study echoes the need to conduct high-quality psychopharmacological studies in people with AN. There are severe major limitations in the quality of the studies included in our meta-analysis. Future psychopharmacological studies for AN need to be large-scale and involve multiple centres while including qualitative feedback from patients and patient-related outcomes to address these limitations. Involving patients and their families in the co-design process will potentially lead to better engagement and lower dropout rates. Overall, patient-related factors may explain drop-out rates in psychopharmacological trials even better than drug-related factors and side effects.

## Author Contributions

All authors were involved in the conception of the study. CK and JT designed the protocol for this analysis. CK performed the statistical analysis. CK and LE wrote the manuscript. All authors reviewed/edited the manuscript.

## Funding

CK, JT, and HH are part funded by the NIHR Mental Health Biomedical Research Centre at South London and Maudsley NHS Foundation Trust and King's College London. CK has also previously received salary support from Novo Nordisk UK Research Foundation and Marie Curie Fellowship. The views expressed are those of the authors and not necessarily those of the NHS, the NIHR or the Department of Health.

## Conflict of Interest

The authors declare that the research was conducted in the absence of any commercial or financial relationships that could be construed as a potential conflict of interest.

## Appendix 1 Exhaustive list of keywords for search

**Table:**

1. ‘anorexi*’ OR ‘anorexia nervosa’
2. ‘atypical antipsychotic’ OR ‘atypical neuroleptic’ OR ‘second generation antipsychotic’ OR ‘second generation neuroleptic’ OR amisulpride OR aripiprazole OR asenapine OR brexpiprazole OR cariprazine OR clozapine OR lurasidone OR ‘lurasidone hydrochloride’ OR olanzapine OR paliperidone OR ‘paliperidone palmitate’ OR primavenserin OR quetiapine OR ‘quetiapine fumarate’ OR remoxipride OR risperidone OR sertindole OR ziprasidone.
3. metaboli* OR BMI OR ‘body mass index’ OR obesity OR hba1c OR ‘blood pressure’ OR hypertension OR cholesterol OR QTc OR prolactin OR hyperglycemi* OR diabetes OR ‘weight gain’ OR ‘weight increase’ OR ‘metabolic side effects of drugs and substances’ OR ‘metabolic diseases’ OR ‘metabolic syndrome’ OR ‘weight gain — drug effects’ OR ‘metabolic networks and pathways — drug effects’ OR ‘body weight — drug effects
4. 1 AND 2 AND 3
5. Limit 4 to humans + English language

## References

[1] Steinhausen H-C The Outcome of Anorexia Nervosa in the 20th Century. Am J Psychiatry (2002) 159:1284–93. 10.1176/appi.ajp.159.8.1284 12153817

[2] Eddy KT Tabri N Thomas JJ Murray HB Keshaviah A Hastings E Recovery From Anorexia Nervosa and Bulimia Nervosa at 22-Year Follow-Up. J Clin Psychiatry (2017) 78:184–9. 10.4088/JCP.15m10393 PMC7883487 28002660

[3] Holland J Hall N Yeates DGR Goldacre M Trends in hospital admission rates for anorexia nervosa in Oxford (1968-2011) and England (1990-2011): database studies. J R Soc Med (2016) 109:59–66. 10.1177/0141076815617651 26609127 PMC4793765

[4] NICE National Clinical Practice Guideline: eating disorders: core interventions in the treatment and management of anorexia nervosa, bulimia nervosa, and related eating disorders. Natl Inst Clin Excell (2004) 9:2015. 23346610

[5] NICE (2018). Eating disorders: recognition and treatment., Available at: https://www.nice.org.uk/guidance/ng69.

[6] Murray SB Quintana DS Loeb KL Griffiths S Le Grange D Treatment outcomes for anorexia nervosa: a systematic review and meta-analysis of randomized controlled trials. Psychol Med (2018), 49(4):535–44. 10.1017/S0033291718002088 30101734

[7] Hay PJ Touyz S Claudino AM Lujic S Smith CA Madden S Inpatient versus outpatient care, partial hospitalisation and waiting list for people with eating disorders. Cochrane Database Syst Rev (2019) 1:CD010827. 10.1002/14651858.CD010827.pub2 30663033 PMC6353082

[8] Beykloo M Y Nicholls D Simic M Brauer R Mills E Wong ICK Survey on self-reported psychotropic drug prescribing practices of eating disorder psychiatrists for the treatment of young people with anorexia nervosa. BMJ Open (2019) 9:e031707. 10.1136/bmjopen-2019-031707 PMC6756572 31542765

[9] Kishi T Kafantaris V Sunday S Sheridan EM Correll CU Are antipsychotics effective for the treatment of anorexia nervosa? Results from a systematic review and meta-analysis. J Clin Psychiatry (2012) 73:e757–66. 10.4088/JCP.12r07691 22795216

[10] Lebow J Sim LA Erwin PJ Murad MH The effect of atypical antipsychotic medications in individuals with anorexia nervosa: a systematic review and meta-analysis. Int J Eat Disord (2013) 46:332–9. 10.1002/eat.22059 23001863

[11] de Vos J Houtzager L Katsaragaki G van de Berg E Cuijpers P Dekker J Meta analysis on the efficacy of pharmacotherapy versus placebo on anorexia nervosa. J Eat Disord (2014) 2:27. 10.1186/s40337-014-0027-x 25379181 PMC4221720

[12] Dold M Aigner M Klabunde M Treasure J Kasper S Second-generation antipsychotic drugs in anorexia nervosa: a meta-analysis of randomized controlled trials. Psychother Psychosom (2015) 84:110–6. 10.1159/000369978 25722106

[13] Chaplain S Taylor M Drug points: second generation antipsychotics. Prescriber (2014) 14:12–21. 10.1002/psb.1269

[14] Watson HJ Yilmaz Z Thornton LM Hübel C Coleman JRI Gaspar HA Genome-wide association study identifies eight risk loci and implicates metabo-psychiatric origins for anorexia nervosa. Nat Genet (2019) 51:1207–14. 10.1038/s41588-019-0439-2 PMC6779477 31308545

[15] Himmerich H Joaquim M Bentley J Kan C Dornik J Treasure J Psychopharmacological options for adult patients with anorexia nervosa: the patients' and carers' perspectives. CNS Spectr (2017) (4):251–52. 10.1017/S1092852917000529 28870274

[16] Cuijpers P Targets and outcomes of psychotherapies for mental disorders: an overview. World Psychiatry (2019) 18:276–85. 10.1002/wps.20661 PMC6732705 31496102

[17] Higgins JPT Altman DG Gotzsche PC Juni P Moher D Oxman AD The Cochrane Collaboration's tool for assessing risk of bias in randomised trials. BMJ (2011) 343:d5928–8. 10.1136/bmj.d5928 PMC3196245 22008217

[18] Morgan RL Thayer KA Santesso N Holloway AC Blain R Eftim SE Evaluation of the risk of bias in non-randomized studies of interventions (ROBINS-I) and the ‘target experiment' concept in studies of exposures: Rationale and preliminary instrument development. Environ Int (2018) 120:382–7. 10.1016/j.envint.2018.08.018 PMC9581061 30125855

[19] Viechtbauer W Conducting meta-analyses in R with the metafor package. J Stat Softw (2010) 36:1–48. 10.18637/jss.v036.i03

[20] Barendregt JJ Doi SA Lee YY Norman RE Vos T Meta-analysis of prevalence. J Epidemiol Community Health (2013) 67:974–8. 10.1136/jech-2013-203104 23963506

[21] Freeman MF Tukey JW Transformations related to the angular and the square root. Ann Math Stat (1950) 21:607–11. 10.1214/aoms/1177729756

[22] Miller J The inverse of the Freeman-Tukey double arcsine transformation. Am Stat (1978) 32:138. 10.2307/2682942

[23] Cochran WG The comparison of percentages in matched samples. Biometrika (1950) 37:256–66. 10.1093/biomet/37.3-4.256 14801052

[24] Begg CB Mazumdar M Operating characteristics of a rank correlation test for publication bias. Biometrics (1994) 50:1088–101. 10.2307/2533446 7786990

[25] Brambilla F Garcia CS Fassino S Daga GA Favaro A Santonastaso P Olanzapine therapy in anorexia nervosa: psychobiological effects. Int Clin Psychopharmacol (2007) 22:197–204. 10.1097/YIC.0b013e328080ca31 17519642

[26] Brambilla F Monteleone P Maj M Olanzapine-induced weight gain in anorexia nervosa: involvement of leptin and ghrelin secretion? Psychoneuroendocrinology (2007) 32:402–6. 10.1016/j.psyneuen.2007.02.005 17395395

[27] Bianco G Claps M Marinucci S Montecchi F Use of risperidone in adolescent anorexia nervosa. Ital J Psychiatry Behav Sci (2000) 10:50–2.

[28] Attia E Steinglass JE Walsh BT Wang Y Wu P Schreyer C Olanzapine Versus Placebo in Adult Outpatients With Anorexia Nervosa: A Randomized Clinical Trial. Am J Psychiatry (2019) 176:449–56. 10.1176/appi.ajp.2018.18101125 PMC7015155 30654643

[29] Spettigue W Norris ML Maras D Obeid N Feder S Harrison ME Evaluation of the Effectiveness and Safety of Olanzapine as an Adjunctive Treatment for Anorexia Nervosa in Adolescents: An Open-Label Trial. J Can Acad Child Adolesc Psychiatry (2018) 27:197–208. 30038658 PMC6054282

[30] Frank GK Shott ME Hagman JO Schiel MA DeGuzman MC Rossi B The partial dopamine D2 receptor agonist aripiprazole is associated with weight gain in adolescent anorexia nervosa. Int J Eat Disord (2017) 50:447–50. 10.1002/eat.22704 PMC5392387 28334444

[31] Marzola E Desedime N Giovannone C Amianto F Fassino S Abbate-Daga G Atypical antipsychotics as augmentation therapy in anorexia nervosa. PloS One (2015) 10:e0125569. 10.1371/journal.pone.0125569 25922939 PMC4414549

[32] Powers PS Klabunde M Kaye W Double-blind placebo-controlled trial of quetiapine in anorexia nervosa. Eur Eat Disord Rev (2012) 20:331–4. 10.1002/erv.2169 PMC3755505 22535517

[33] Attia E Kaplan AS Walsh BT Gershkovich M Yilmaz Z Musante D Olanzapine versus placebo for out-patients with anorexia nervosa. Psychol Med (2011) 41:2177–82. 10.1017/S0033291711000390 21426603

[34] Hagman J Gralla J Sigel E Ellert S Dodge M Gardner R A double-blind, placebo-controlled study of risperidone for the treatment of adolescents and young adults with anorexia nervosa: a pilot study. J Am Acad Child Adolesc Psychiatry (2011) 50:915–24. 10.1016/j.jaac.2011.06.009 PMC3171450 21871373

[35] Kafantaris V Leigh E Hertz S Berest A Schebendach J Sterling WM A Placebo-Controlled Pilot Study of Adjunctive Olanzapine for Adolescents with Anorexia Nervosa. J Child Adolesc Psychopharmacol (2011) 21:207–12. 10.1089/cap.2010.0139 21663423

[36] Norris ML Spettigue W Buchholz A Henderson KA Gomez R Maras D Olanzapine Use for the Adjunctive Treatment of Adolescents with Anorexia Nervosa. J Child Adolesc Psychopharmacol (2011) 21:213–20. 10.1089/cap.2010.0131 PMC3111870 21510781

[37] Court A Mulder C Kerr M Yuen HP Boasman M Goldstone S Investigating the effectiveness, safety and tolerability of quetiapine in the treatment of anorexia nervosa in young people: a pilot study. J Psychiatr Res (2010) 44:1027–34. 10.1016/j.jpsychires.2010.03.011 20447652

[38] Leggero C Masi G Brunori E Calderoni S Carissimo R Maestro S Low-Dose Olanzapine Monotherapy in Girls with Anorexia Nervosa, Restricting Subtype: Focus on Hyperactivity. J Child Adolesc Psychopharmacol (2010) 20:127–33. 10.1089/cap.2009.0072 20415608

[39] Bissada H Tasca GA Barber AM Bradwejn J Olanzapine in the Treatment of Low Body Weight and Obsessive Thinking in Women With Anorexia Nervosa: A Randomized, Double-Blind, Placebo-Controlled Trial. Am J Psychiatry (2008) 165:1281–8. 10.1176/appi.ajp.2008.07121900 18558642

[40] Bosanac P Kurlender S Norman T Hallam K Wesnes K Manktelow T An open-label study of quetiapine in anorexia nervosa. Hum Psychopharmacol Clin Exp (2007) 22:223–30. 10.1002/hup.845 17487935

[41] Powers PS Bannon Y Eubanks R McCormick T Quetiapine in anorexia nervosa patients: an open label outpatient pilot study. Int J Eat Disord (2007) 40:21–6. 10.1002/eat.20325 16927383

[42] Mondraty N Birmingham CL Touyz S Sundakov V Chapman L Beumont P Randomized controlled trial of olanzapine in the treatment of cognitions in anorexia nervosa. Australas Psychiatry (2005) 13:72–5. 10.1080/j.1440-1665.2004.02154.x 15777417

[43] Barbarich NC McConaha CW Gaskill J La Via M Frank GK Achenbach S An open trial of olanzapine in anorexia nervosa. J Clin Psychiatry (2004) 65:1480–2. 10.4088/JCP.v65n1106 15554759

[44] Malina A Gaskill J McConaha C Frank GK LaVia M Scholar L Olanzapine treatment of anorexia nervosa: a retrospective study. Int J Eat Disord (2003) 33:234–7. 10.1002/eat.10122 12616591

[45] Powers PS Santana CA Bannon YS Olanzapine in the treatment of anorexia nervosa: an open label trial. Int J Eat Disord (2002) 32:146–54. 10.1002/eat.10084 12210656

[46] Ruggiero GM Laini V Mauri MC Ferrari VM Clemente A Lugo F A single blind comparison of amisulpride, fluoxetine and clomipramine in the treatment of restricting anorectics. Prog Neuropsychopharmacol Biol Psychiatry (2001) 25:1049–59. 10.1016/s0278-5846(01)00174-9 11444677

[47] Beasley CM Sanger T Satterlee W Tollefson G Tran P Hamilton S Olanzapine versus placebo: results of a double-blind, fixed-dose olanzapine trial. Psychopharmacol (Berl) (1996) 124:159–67. 10.1007/bf02245617 8935812

[48] Kemmler G Hummer M Widschwendter C Fleischhacker WW Dropout rates in placebo-controlled and active-control clinical trials of antipsychotic drugs. Arch Gen Psychiatry (2005) 62:1305. 10.1001/archpsyc.62.12.1305 16330718

[49] Linardon J Hindle A Brennan L Dropout from cognitive-behavioral therapy for eating disorders: a meta-analysis of randomized, controlled trials. Int J Eat Disord (2018). 51(5):381–91. 10.1002/eat.22850 29493805

[50] Costa MB Melnik T Effectiveness of psychosocial interventions in eating disorders: an overview of Cochrane systematic reviews. Einstein (Sao Paulo) (2016) 14:235–77. 10.1590/S1679-45082016RW3120 PMC4943360 27462898

[51] Linardon J Fitzsimmons-Craft EE Brennan L Barillaro M Wilfley DE Dropout from interpersonal psychotherapy for mental health disorders: a systematic review and meta-analysis. Psychother Res (2019) 29:870–81. 10.1080/10503307.2018.1497215 30005586

